# Predicted Cold Shock Proteins from the Extremophilic Bacterium* Deinococcus maricopensis* and Related* Deinococcus* Species

**DOI:** 10.1155/2017/5231424

**Published:** 2017-09-18

**Authors:** Michael J. LaGier

**Affiliations:** Department of Biology, Grand View University, Des Moines, IA, USA

## Abstract

While many studies have examined the mechanisms by which extremophilic Deinococci survive exposure to ionizing radiation, very few publications have characterized the cold shock adaptations of this group, despite many species being found in persistent cold environments and environments prone to significant daily temperature fluctuations. Bacterial cold shock proteins (Csps) are a family of conserved, RNA chaperone proteins that commonly play a role in cold temperature adaptation, including a downward shift in temperature (i.e., cold shock). The primary aim of this study was to test whether a representative, desert-dwelling* Deinococcus*,* Deinococcus maricopensis*, encodes Csps as part of its genome. Bioinformatic approaches were used to identify a Csp from* D. maricopensis *LB-34. The Csp, termed Dm-Csp1, contains sequence features of Csps including a conserved cold shock domain and nucleic acid binding motifs. A tertiary model of Dm-Csp1 revealed an anticipated Csp structure containing five anti-parallel beta-strands, and ligand prediction experiments identified N-terminally located residues capable of binding single-stranded nucleic acids. Putative Csps were identified from 100% of (27 of 27) Deinococci species for which genome information is available; and the Deinococci-encoded Csps identified contain a C-terminally located region that appears to be limited to members of the class Deinococci.

## 1. Introduction

The Genus* Deinococcus *currently contains 59 recognized species (http://www.bacterio.net), including 27 species for which genomic information is available (https://www.ncbi.nlm.nih.gov/genome/browse/). Members of* Deinococcus *demonstrate a capacity to resist environmental stress including exposure to significant levels of ionizing radiation and desiccation [1]. Species of Deinococci have been isolated from a wide range of environments including air, desert soil, fresh and marine waters, alpine environments, Antarctica, hot springs, and radioactive sites [2]. One species,* Deinococcus maricopensis*, was originally isolated from an arid soil sample collected from the Sonoran Desert in Arizona (USA), and the genome of the type strain of this species (LB-34) has been recently sequenced as part of the Genomic Encyclopedia of Bacteria and Archaea project [3, 4].* D. maricopensis *LB-34 cells are mesophilic, Gram-positive, rod-shaped, and nonmotile, with an optimal growth temperature of 40°C and an observed growth range of 10°C to 45°C [3]. The relatively wide growth range of* D. maricopensis *correlates with observed diurnal swings of 15°C or more during a typical day within the Sonoran Desert (https://science.nature.nps.gov/im/units/sodn/sonoran.cfm). Hence,* D. maricopensis *has adapted to an environment prone to rapid temperature changes, including a shift to colder temperatures.

Although many studies have examined the mechanisms by which Deinococci survive exposure to significant levels of ionizing radiation, few studies have looked at the cold shock adaptations of this group, despite many species being found in persistent cold environments (e.g., Antarctica, cold water oceans) and environments prone to significant daily temperature fluctuations such as deserts [5–7]. Using the model species of the group,* Deinococcus radiodurans, *data from a previous proteomic study showed changes in global protein expression patterns following a cold shock [8]. Although this study was useful in demonstrating that gene expression changes do occur in response to cold shock, studies are lacking that examine additional Deinococci genomes for putative cold shock proteins.

Bacterial cold shock proteins (Csps) represent a family of small proteins (smaller than 100 amino acids in length) with common structural features and a capacity to bind to single-stranded nucleic acids by way of conserved, N-terminally located nucleic acid-binding motifs [9]. Csp expression levels are typically increased following an ambient temperature decrease, but not always [9, 10]. Within the Deinococci species, the best characterized protein that shares significant sequence homology to known Csps is from* D. radiodurans *[11]. The protein, termed PprM, appears to play a role in the radiation and oxidative stress resistance phenotypes of* D. radiodurans *[11]. Interestingly, while one study showed PprM to be induced [8] after heat shock (and apparently not induced after cold shock), a second study failed to demonstrate induction of PprM after heat shock [12]. Hence, our understanding of the potential role of Csps within the Deinococci species as a group is limited.

Outside the Deinococci species, the best studied Csps come from the mesophile* Escherichia coli*, although putative Csps have been identified from psychrophiles, mesophiles, and thermophiles [13]. In* E. coli, *nine members of the Csp family have been identified: CspA, B, C, D, E, F, G, H, and I. Although a significant number of the* E. coli *Csps are induced by cold (A, B, G, and I), E and C are constitutively expressed at physiological temperature, and D is induced via nutrient stress [9]. Of the nine, CspA is best characterized and appears to play a role in bacterial cold shock by acting as an RNA chaperone, destabilizing secondary structures within mRNAs that are more likely to form as extracellular temperatures decrease, and thus can reduce transcriptional and translational efficiency [9, 13, 14].

The current study identifies and characterizes, using bioinformatics, a potential Csp homolog from* D. maricopensis*, a desert-dwelling species that is naturally exposed to significant temperature fluctuations including daily temperature downshifts [3]. The* D. maricopensis *Csp, termed Dm-Csp1, contains expected nucleic acid binding motifs, shares significant sequence identity and similarity to known Csp proteins, contains five conserved anti-parallel beta-strands according to structural modeling, and is predicted to have a capacity to bind single-stranded nucleic acids. In addition, the presence of Csps was identified in all Deinococci genomes queried (27 of 27). Interestingly, the Csps identified all contain a C-terminal region that is unique among members of the class deinococci; and for which a potential function is unknown.

## 2. Materials and Methods

### 2.1. Sequence Retrieval

Protein data available for* Deinococcus maricopensis *LB-34 at the National Center for Biotechnology Information (NCBI) was queried via a text search using “cold shock” as the input. An 86 amino acid long protein, NCBI Accession #WP 013556416.1 was then selected for further study by storing the protein sequence locally as a FASTA file. This protein was termed Dm-Csp1.

### 2.2. Analysis of Primary Features of Dm-Csp1

A tool within the ExPASy site, ProtParam (http://web.expasy.org/protparam/), was used to predict the basic physiological and chemical features of Dm-Csp1. For comparison, a known Csp protein (CspA) sequence from* E. coli *K12 (NCBI Accession #P0A9X9) was subjected to ProtParam analysis in parallel. Default settings were used for ProtParam analyses. ProtParam predicts properties of input protein sequences including molecular weight, isoelectric pH, aliphatic index, and extinction coefficients.

### 2.3. Sequence Similarity Analyses of Dm-Csp1

The FASTA protein sequence of Dm-Csp1 was used as a query in standard BLASTp searches against the nonredundant database (nr) as well as the PDB protein database, which includes only sequences for which a solved protein structure is available (http://www.rcsb.org/pdb/home/home.do). To identify potential Csp homologs in the Deinococci as a group, a standard BLASTp search was run (using Dm-Csp1 as the query) for which the nr database was limited to sequences from members of the Genus* Deinococcus* (27 species total, including* D. maricopensis*) containing permanent draft or finished genomic data available at the Integrated Microbial Genomes and Microbiomes (IMG/M) site (https://img.jgi.doe.gov/). A combination of Pfam and ScanProsite was used to identify conserved protein domains within the sequence of Dm-Csp1, under default settings [15, 16]. To determine the number and type of Csps in* D. maricopensis*, CspA, B, C, D, E, F, G, H, and I protein sequences from* E. coli *K12 were used via BLASTp to query the genome of* D. maricopensis *LB-34. Unless stated otherwise, BLASTp was run according to default parameters.

### 2.4. Multiple Sequence Alignment

**Figure 1:**
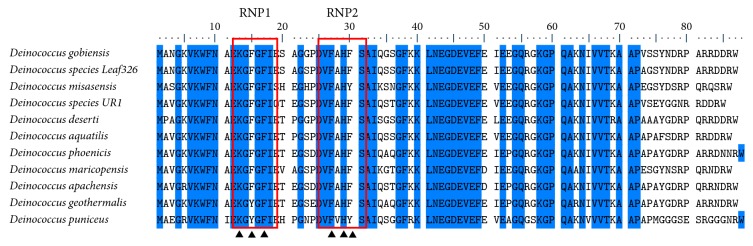
*Multiple sequence alignment of Dm-Csp1 and related, representative Deinococci-encoded cold shock proteins*. The species names are indicated. Areas of 100% conservation are highlighted in blue. The position of conserved RNA-binding motifs (RNP1 and RNP2) is enclosed within red rectangles. Conserved, basic, and aromatic residues within RNP1 and RNP2 that form a nucleic acid binding surface are indicated by arrowheads.

BLASTp, with Dm-Csp1 as a query, was used to identify closely related proteins from within the Genus Deinococcus. Dm-Csp1, along with 10 putative Csp proteins from 10 representative Deinococci, was retrieved as FASTA files from NCBI and aligned using standard parameters of the BioEdit Sequence Alignment Editor (http://www.mbio.ncsu.edu/bioedit/bioedit.html). Amino acids 100% conserved (Figure 1) or 50% conserved (Figure 4) among the 11 aligned proteins were highlighted in blue, and functional motifs were identified by visual inspection of the output alignment.

### 2.5. Protein to Protein Interactions

The STRING tool was used to identify proteins that Dm-Csp1 may interact with* in vivo*. The STRING database contains information from experimental data and public text collections to predict protein-protein interactions [17]. The basic interaction unit in STRING is the functional association, likely contributing to a common biological purpose. Predicted protein-protein interactions are derived from multiple sources including known experimental interactions (coexpression, experiments), genome functional pathway knowledge from manually curated databases (databases), automated text-mining searches (text-mining), shared gene neighborhood locations across multiple genomes (neighborhood), and interactions observed in one organism and systematically transferred to other organisms, via precomputed orthology relations (cooccurrence and gene fusion). Default parameters of STRING were used.

### 2.6. Dm-Csp1 Structure Prediction and Model Quality Assessment

The secondary structure of Dm-Csp1 was predicted by the PSIPRED server (http://bioinf.cs.ucl.ac.uk/psipred/), under default settings. The tertiary structure of Dm-Csp1 was modeled using the (PS)^2^-v2 server [18]. In (PS)^2^-v2, autodetect functionality was employed to select a suitable template structure to model against. The output model of Dm-Csp1 was visualized by Chimera software [19]. The quality of the predicted structure was assessed by PROCHECK [20], QMEAN6 [21], and verify3D [22]. PROCHECK and QMEAN6 are programs within the SWISS-MODEL workspace (https://swissmodel.expasy.org), and verify3D is found at the UCLA-DOE Structure Evaluation Server (http://services.mbi.ucla.edu/Verify_3D/).

### 2.7. Dm-Csp1 Ligand Binding Site Prediction

The model file generated from (PS)^2^-v2 was used as an input into COACH [23] to predict potential ligand binding regions within Dm-Csp1. COACH is a metaserver approach to protein ligand binding site prediction. COACH generates predictions using TM-SITE and S-SITE, which recognize ligand binding templates from the BioLiP protein function database by binding specific substructure and sequence profile comparisons. These predictions are combined with predictions from COFACTOR, FINDSITE, and ConCavity to generate final ligand binding site predictions [23].

## 3. Results and Discussion

### 3.1. Identification of Csps from* D. maricopensis* and Related Deinococci

Initial text queries of* D. maricopensis *protein sequences available at NCBI identified a potential protein of 86 amino acids in length denoted as a cold shock protein (Csp). The protein, designated in this study as Dm-Csp1 (NCBI Accession #WP 013556416.1), was then used as a query sequence in additional BLASTp searches. Specifically, BLASTp analysis against the nonredundant (nr) and PDB protein database showed that Dm-Csp1 shares significant sequence identity and similarity to bacterial proteins annotated as Csps, including those for which a protein structure has been solved (Table 1). In addition, predicted physiological and chemical features of Dm-Csp1 were similar to those obtained when compared to a known Csp from the model bacterium* E. coli*. As shown in Table 2, Dm-Csp1 is predicted, according to ProtParam, to have a molecular mass of 7.4 Kilodaltons and an isoelectric pH of 5.58.

To better understand the conservation of Csps among the Genus* Deinococcus*, the sequence of Dm-Csp1 was used as a BLASTp query to scan the genomes of related Deinococci species, for which genomic sequence information is available (27 total species, including* D. maricopensis*). As summarized in Table 3, at least one protein sharing significant sequence identity and similarity (as well as significant *E*-values), was identified in 100% of the Deinococci species queried. Although the number of Csps per bacterial genome can vary according to species, the reasoning behind such variation is unknown [10, 13]. The absolute conservation of Csps among the Deinococci sampled implies that Csps may be a significant feature of this groups' capacity in adapting to cold shock conditions and/or adapting or responding to other stress conditions.

While* E. coli *has been shown to contain a total of 9 Csps per genome, the* D. maricopensis *LB-34 genome appears to contain 2 Csps, according to BLAST testing (Section 2). Specifically, in addition to Dm-Csp1, the LB-34 genome appears to contain a second Csp, termed in this study as Dm-Csp2 (NCBI Accession #WP 013556476.1), also 86 amino acids in total length. In comparison to* E. coli *Csps (A–I), Dm-Csp1 shares the greatest homology with* E. coli *CspA (BLASTp *E*-value of 9.0*E* − 23) and Dm-Csp2 shares greatest homology with CspB (BLASTp *E*-value of 2.0*E* − 21). Dm-Csp1 and Dm-Csp2 share 95% sequence identity and 96% sequence similarity. Dm-Csp1 and Dm-Csp2 differ in a total of 4 amino acids, none of which are found within key, conserved regions of known Csps (data not shown). Hence, Dm-Csp1 and Dm-Csp2 may serve similar functions in* D. maricopensis*. Within a single genome, it is anticipated that bacterial Csps share significant levels of sequence identity and similarity, as is the case with Csps from* E. coli *and* Yersinia pseudotuberculosis* [9]. In* E. coli *and* Y. pseudotuberculosis*, having more than one Csp sharing significant sequence homology is thought to represent functional redundancy, as mutagenesis of one or more Csps can be functionally compensated by remaining, nonmutated homologs [24, 25].

In addition to sequence homology testing, the primary sequence of Dm-Csp1 was compared to a sample of Deinococci-encoded Csps by multiple sequence alignments and was also used as a query to identify conserved protein domains. Pfam searching identified a conserved cold shock DNA-binding domain (Clan #CL0021) within the N-terminal region of Dm-Csp1 with a significant *E*-value score of 2.9*E* − 25. Consistent with Pfam, ScanProsite results indicated an N-terminally located cold shock domain signature (#PS00352) from amino acid position 15 to 33 with good confidence. A multiple sequence of Dm-Csp1 with 10 related Deinococci-encoded Csps revealed the presence of an additional feature of Csps, two nonspecific RNA-binding sequence motifs [9], ribonucleotide motifs 1 and 2 (Figure 1, RNP1 and RNP2). In bacterial Csps, the canonical sequence for RNP1 is (K/S-G-F/K/Y-G-F/L-I) and for RNP2 is (L/I/V-F/Q-V/A/L-H-X-S/T/R). In the aligned representative Deinococci (Figure 1) Csps, the sequences of RNP1 and RNP2 are very similar to expected, with RNP1 being (K-G-F/Y-G-F-I-X-X) and RNP2 being (V-F-V/A-H-F/Y-S). Within the known tertiary structures of Csps, the basic and aromatic residues within RNP1 and RNP2, collectively, form a nucleic acid-binding surface (Figure 1).

### 3.2. Structural Analyses of Dm-Csp1

**Figure 2:**
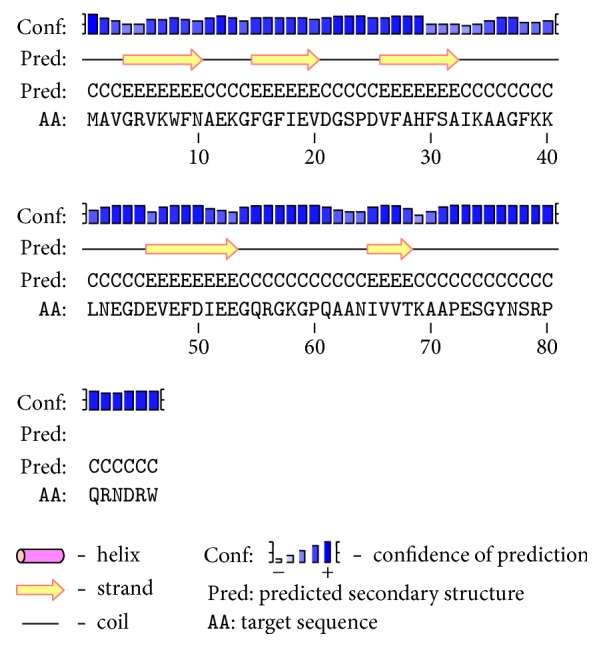
*Secondary protein structure of Dm-Csp1 as predicted by PSIPRED*. The relative height of blue bars indicates confidence of prediction.

To better understand the potential biological function of Dm-Csp1, a structural model of Dm-Csp1 was constructed. The secondary structure of Dm-Csp1 was predicted using the PSIPRED server. The result of PSIPRED, as shown in Figure 2, has good confidence and contains 5 beta-strands, which are expected among Csps [9, 26].

**Figure 3:**
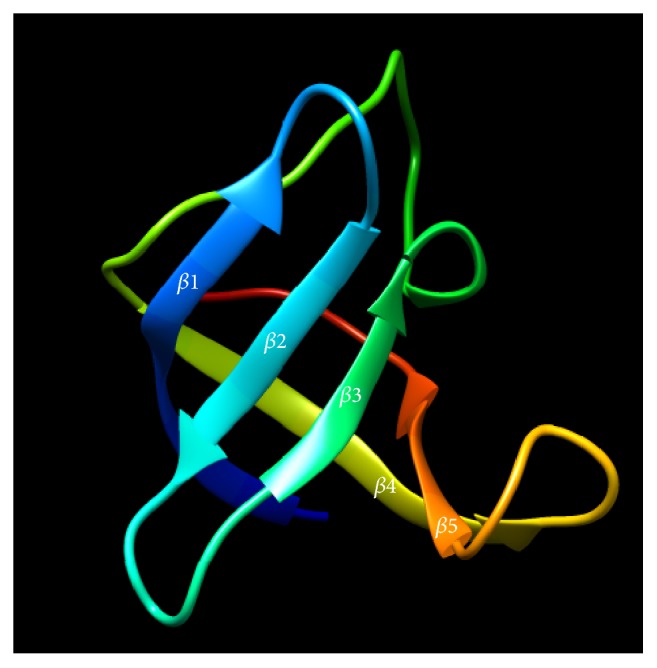
*Modeled tertiary structure of Dm-Csp1*. The five anti-parallel strands that collectively form a barrel structure are indicated.

**Figure 4:**
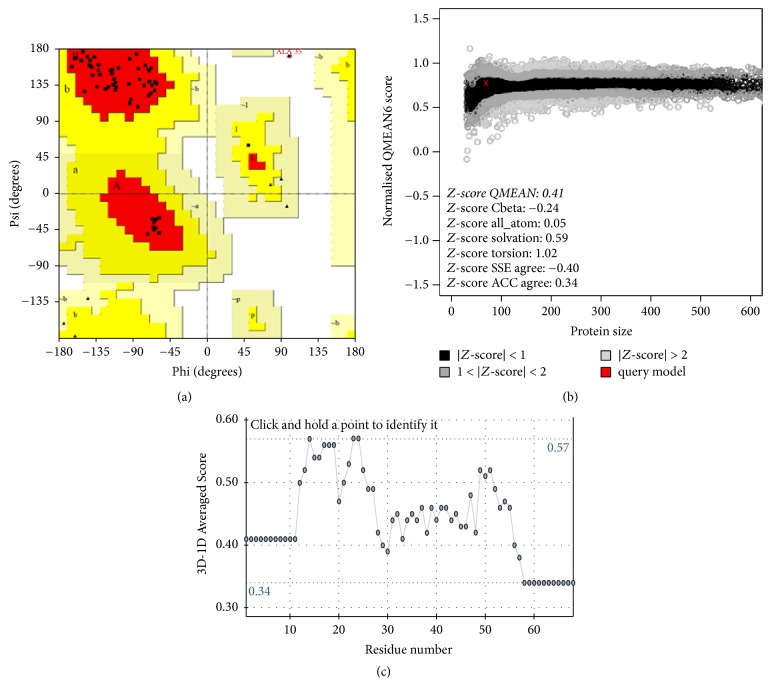
*Tertiary structural model assessment*. (a) Ramachandran plot of protein model generated by PROCHECK. The position of 96.4% amino acids within the generated structural model are within most favored regions. The position of 1 amino acid (ALA-35, red text) is found within a disallowed region. (b) Graphical output of estimation of absolute quality of model via QMEAN6. The position of the Dm-Csp1 tertiary model is indicated by a red X. (c) Verify3D output data for Dm-Csp1 predicted tertiary structure, yielding an acceptably high score of 0.57.

The modeling of the likely tertiary structure of Dm-Csp1, as predicted by (PS)^2^-v2, used a known Csp from* Bacillus caldolyticus *(PDB #1C9O) as a template. The* B. caldolyticus *Csp crystal structure used as a template is 66 amino acids in length and shares 61.76% sequence identity with Dm-Csp1 [27]. The model of Dm-Csp1 was found to contain five antiparallel beta-strands that form a barrel structure (Figure 3). These features are known characteristics of Csps. The quality of the output model structure was assessed using a combination of PROCHECK, QMEAN6, and verify3D (Section 2). PROCHECK reveals that all amino acids residues within the model (68 of 68) are within the limits of a Ramachandran plot, with 96.4% of residues in the most favored regions and only a single residue (1.8%) in a disallowed region (Figure 4(a)). A good model is expected to have over 90% of residues in most favored regions [20]. QMEAN6, which estimates the global quality of structural models on the basis of 6 structural descriptors, places the model of Dm-Csp1 in the dark region of the estimated absolute model quality graph with a significant QMEAN6 score of 0.781 and a significant *Z*-score of 0.413 (Figure 4(b)). Using verify3D, the environmental profile of the model was acceptable (Figure 4(c)), yielding a high score of 0.57 [22].

**Figure 5:**
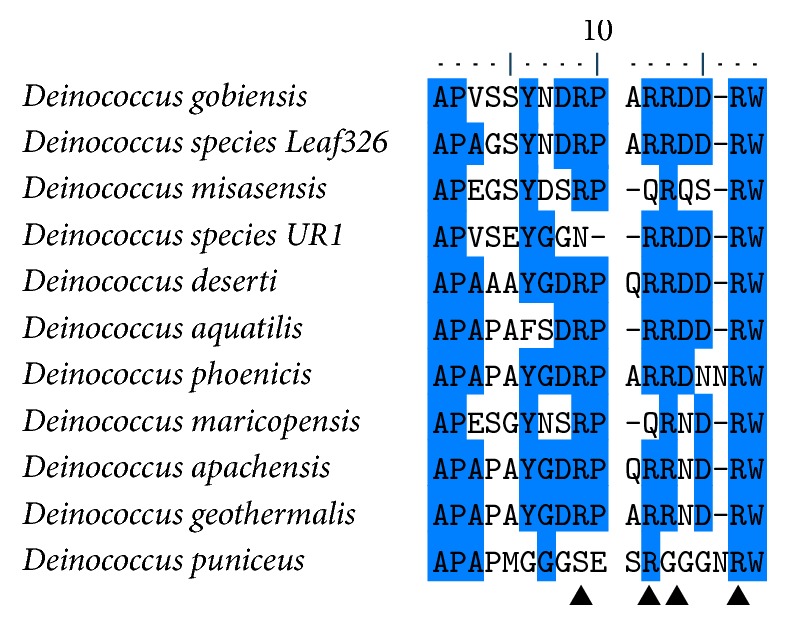
*Multiple sequence alignment of C-terminal region of Dm-Csp1 and related, representative Deinococci-encoded cold shock proteins*. Species names are indicated, regions of 50% or greater conservation are marked blue, and the positions of arginine-rich areas are denoted by arrowheads.

The C-terminal region of Dm-Csp1 could not be modeled, specifically from amino acids 69 to 86. The reason for this inability to model is due to the overall length of Dm-Csp1 being 86 amino acids. Bacterial Csps identified thus far are approximately 70 amino acids in length, with the N-terminal region containing conserved nucleic acid binding regions [9, 13]. The extended C-terminal region of Dm-Csp1, according to BLASTp analysis, appears to be unique to the class Deinococci. For example, in addition to species of Deinococci, some members (for which genomic information is available) of the class Deinococci encode at least one putative Csp with the C-terminal extension including* Truepera radiovictrix *(data not shown, NCBI Accession #WP_013178602). As shown in Figure 5, all the Deinococci Csps included in the Figure 1 alignment contain an extended C-terminal region that appears to be rich in positively charged arginine residues and end in the combination arginine-tryptophan. The potential function of this conserved region within Deinococci Csps is unknown but warrants further investigation.

### 3.3. Biological Function Prediction

**Figure 6:**
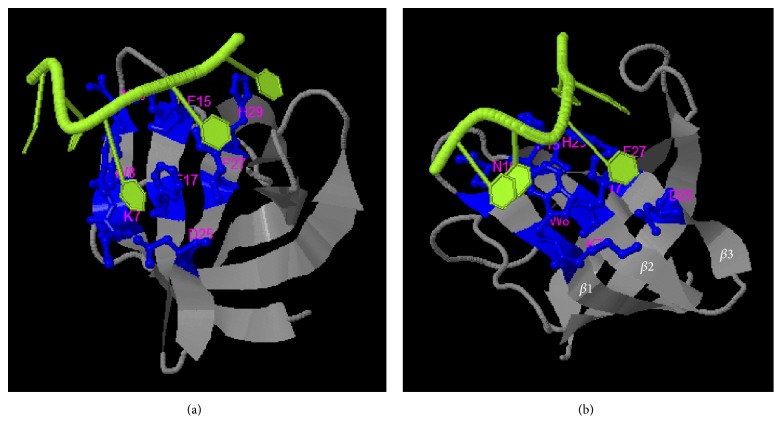
*Functional site prediction of Dm-Csp1*. COACH output identifying N-terminally located residues predicted to bind single-stranded nucleic acids ((a), nucleic acid colored green, binding residues colored blue), and the same output repositioned to show the predicted nucleic acid binding residues is found within three anti-parallel beta-strands that collectively form a nucleic acid binding surface (b).

**Figure 7:**
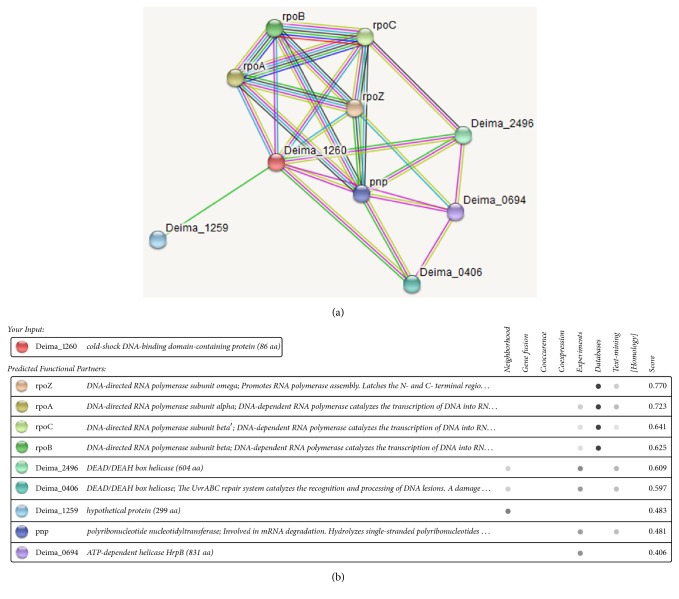
*STRING protein-protein interactions of Dm-Csp1*. STRING network map (a) and summary table of predicted interactions (b). The category of interaction is shown in the table. Color saturation of the edges represents the confidence score of a given functional or physical association. The position of Dm-Csp1 is labeled Deima_1260. Shown interactions are considered of high confidence when yielding scores of greater than 0.7.

The synthesized, and quality assessed, structural model of Dm-Csp1 was used as a template to predict the potential of the protein to bind ligands. Using the COACH server, the N-terminal region of Dm-Csp1 appears able to bind single-stranded nucleic acids, with a significant support score (*C*-score) of 0.2 [23]. A total of 8 residues are predicted to bind nucleic acids, 4 of which are located with the previously identified within the RNP1 and RNP2 motifs including F15, F17, F27, and H29 (Figure 6(a)). The binding of nucleic acids using a combination of three sequential beta-strands (Figure 6(b)) is consistent with previously solved structures of Csps [9, 13, 14, 26, 28]. According to STRING analysis, Dm-Csp1 is predicted to functionally interact with a variety of proteins including those (rpoB, rpoC, and rpoA, and rpoZ, RNA polymerase subunits) involved in transcription and a polyribonucleotide nucleotidyltransferase (pnp) potentially playing a role in mRNA degradation (Figure 7). The predicted interactions of Dm-Csp1 with transcription-related proteins make sense when considering previous studies demonstrating the role of Csps in modulating transcription, including acting as a RNA chaperone [9, 13, 29, 30].

## 4. Conclusions

The findings here suggest that one adaptation by which* D. maricopensis *deals with significant, diurnal temperature shifts is by utilizing genome-encoded cold shock proteins (Csps). Significantly, all additional members of the Genus Deinococcus, for which genomic information is available, also encode Csps. Interestingly, the identified Deinococci Csps contain a C-terminal extension not observed in bacteria outside the class Deinococci, and for which a function is undefined. Future studies will seek to further characterize the biological roles of Dm-Csp1, and related Csps, via* in vitro *approaches.

## Figures and Tables

**Table 1:** BLASTp analysis of Dm-Csp1 (NCBI Accession #WP 013556416.1).

Database identifier^a^	BLASTp hit species	Name of hit	GenBank hit number	*E*-value	Identity (%)	Similarity (%)	Hit query coverage (%)
nr	*Deinococcus maricopensis*	Cold shock protein	WP 013556476.1^b^	5.00*E* − 56	95	96	100
nr	*Deinococcus peraridilitoris*	Cold shock protein	WP 015234625.1	2.00*E* − 48	84	90	100
nr	*Deinococcus aqualitis*	Cold shock protein	WP 019008855.1	7.00*E* − 48	80	91	100
nr	*Deinococcus misasensis*	Cold shock protein	WP 034335902.1	1.00*E* − 47	80	88	100
nr	*Deinococcus pimensis*	Cold shock protein	WP 027481785.1	2.00*E* − 47	83	87	100
PDB	*Bacillus caldolyticus*	Cold shock protein	115F_A^c^	6.00*E* − 24	64	75	76
PDB	*Bacillus caldolyticus*	Cold shock protein	1C90_A	6.00*E* − 24	66	75	76
PDB	*Bacillus subtilis*	Cold shock protein	2ES2_A	6.00*E* − 24	64	73	82
PBD	*Bacillus caldolyticus*	Cold shock protein	1HZB_A	6.00*E* − 24	64	75	76
PDB	*Bacillus subtilis*	Cold shock protein	2I5L_X	8.00*E* − 24	66	73	74

^a^Nonredundant database (nr) or protein database (PDB), acquired via independent BLASTp searches using Dm-Csp1 as a query. ^b^Referred to as Dm-Csp2 within text. ^c^Protein database identifier code.

**Table 2:** Physiological and chemical properties of Dm-Csp1 and *E. coli *K12 CspA^a^.

Protein identifier	Amino acid length	Molecular weight	Isoelectric point	Aliphatic index	Estimated half-life	Positively charged residues (%)	Negatively charged residues (%)
Dm-Csp1	86	9472.54	5.79	57.91	>10 hours	13.95	15.12
*E. coli* CspA	70	7403.28	5.58	61.29	>10 hours	10	11.42

^a^Calculated using default settings of ProtParam (http://web.expasy.org/protparam/).

**Table 3:** BLASTp analysis of Dm-Csp1 (NCBI Accession #WP 013556416.1) versus deinococci group.

BLASTp hit species	Name of hit	Gene ID number^a^	*E*-value	Identity (%)	Similarity (%)
*Deinococcus maricopensis*	Cold shock protein	649901717	5.00*E* − 56	95	96
*D. reticulitermitis*	Cold shock protein	2617968483	6.00*E* − 45	76	87
*D. peraridilitoris*	Cold shock protein	2509591723	2.00*E* − 48	84	91
*D. deserti*	Cold shock protein	643799117	7.00*E* − 45	78	85
*D. xibeiensis*	Cold shock protein	2586308013	5.00*E* − 45	77	86
*D. marmoris*	Cold shock protein	2562219581	1.00*E* − 33	72	85
*D. wulumuqiensis*	Cold shock protein	2553983725	5.00*E* − 45	77	86
*D. species *2009	Cold shock protein	2516938626	1.00*E* − 44	78	87
*D. pimensis*	Cold shock protein	2509082093	1.00*E* − 47	83	87
*D. misasensis*	Cold shock protein	2571066521	6.00*E* − 47	79	87
*D. phoenicis*	Cold shock protein	2579632034	1.00*E* − 40	78	84
*D. geothermalis*	Cold shock protein	641229653	2.00*E* − 45	80	87
*D. apachensis*	Cold shock protein	2519136834	3.00*E* − 47	84	87
*D. gobiensis*	Cold shock protein	25128201432	3.00*E* − 47	81	88
*D. proteolyticus*	Cold shock protein	649963862	5.00*E* − 33	62	77
*D. hopiensis*	Cold shock protein	2509586483	2.00*E* − 46	83	86
*D. grandis*	Cold shock protein	2691382520	1.00*E* − 45	80	90
*D. species *RL	Cold shock protein	2611619696	3.00*E* − 47	81	88
*D. radiodurans*	Cold shock protein	2558116098	5.00*E* − 45	77	86
*D. murrayi*	Cold shock protein	2528309711	3.00*E* − 47	81	88
*D. ficus *	Cold shock protein	2526061465	1.00*E* − 33	78	87
*D. fringens*	Cold shock protein	2562223450	1.00*E* − 33	72	85
*D. humi*	Cold shock protein	2597538219	2.00*E* − 47	79	92
*D. soli*	Cold shock protein	2637699313	1.00*E* − 45	80	90
*D. species *Leaf 326	Cold shock protein	2645444050	2.00*E* − 46	82	90
*D. aquatilis*	Cold shock protein	2520600036	7.00*E* − 48	80	92
*D. species *YIM 77859	Cold shock protein	2582523845	8.00*E* − 47	82	91

^a^According to the Integrated Microbial Genomes database (https://img.jgi.doe.gov/).
